# Formulation of α-Linolenic Acid-Based Microemulsions for Age-Related Macular Degeneration: Physicochemical Tests and HET-CAM Assays for Anti-Angiogenic Activities

**DOI:** 10.3390/medicina61112030

**Published:** 2025-11-13

**Authors:** Sang Gu Kang, Mahendra Singh, Gibaek Lee, Kyung Eun Lee, Ramachandran Vinayagam

**Affiliations:** 1 Department of Biotechnology, College of Life and Applied Sciences, Yeungnam University, 280 Daehak-Ro, Gyeongsan 38541, Republic of Korea; m.singh2685@gmail.com (M.S.); eornek2986@ynu.ac.kr (G.L.); 2 Stemforce, 313 Institute of Industrial Technology, Yeungnam University, Gyeongsan 38541, Republic of Korea; stemforce@naver.com

**Keywords:** age-related macular degeneration (AMD), intravitreal injection, geriatric, microemulsion, COX-2 inhibitor, angiogenesis, topical ocular delivery

## Abstract

*Background and Objectives:* Age-related macular degeneration (AMD) is an age-associated retinal disorder characterized by blood–retinal barrier (BRB) breakdown and pathological angiogenesis, leading to vascular leakage. The intravitreal administration of anti-VEGF agents remains the most effective treatment for neovascular AMD. However, repetitive intravitreal injections have risks, causing side effects such as cataracts, bleeding, retina damage, and, in severe cases, post-injection endophthalmitis. Hence, the development of innovative drug delivery systems is essential to minimize the risks and discomfort associated with intravitreal injections. *Materials and Methods:* We developed a microemulsion (ME)-based topical drug delivery system incorporating α-linolenic acid (ALA). In brief, pseudo-ternary phase diagrams were constructed by the water titration method using different combinations of surfactants and cosurfactants (S_mix_-Cremophor RH 40: Span 80: Transcutol P in ratios of 1:1.05, 1:1:1, 1:1:1.5) containing ALA as the oil phase. Three blank microemulsions (ME1, ME2, and ME3) were prepared and characterized based on the optimized pseudo-ternary phase equilibrium with a S_mix_ ratio of 1:1:1. *Results:* ME3, with an average particle size of 38.59 nm, was selected as the optimized formulation for developing drug-loaded ME containing Fenofibrate, Axitinib, and Sirolimus. The drug-loaded ME showed particle size (46.94–56.39 nm) and in vitro release displayed sustained and longer time drug release for 240 h. The irritation and antiangiogenic activities were evaluated using the hen’s egg chorioallantoic membrane (HET-CAM) assay employing the optimized ME loaded with each drug. Among the three drug-loaded ME, the Sirolimus ME showed a reduction in blood vessel sprouting in the HET-CAM assay, indicating strong antiangiogenic activity. Treatment with the optimized blank ME and Sirolimus ME significantly (*p* < 0.05) reduced COX-2 protein expression in LPS-stimulated RAW 264.7 cells, suggesting their potential anti-inflammatory effects. *Conclusions:* Overall, we suggest that the α-linolenic acid-based Sirolimus microemulsion may serve as a promising topical therapeutic approach for managing AMD and offering a potential alternative to invasive intravitreal injections.

## 1. Introduction

Blindness and visual impairment, particularly in the elderly, have a significant negative impact on quality of life by reducing physical mobility, raising the risk of anxiety and depression, and driving up healthcare expenses worldwide.

The vascular leakage or breakdown of the blood–retinal barrier (BRB) can lead to the development of macular edema [1]. Macular edema is the primary cause of age-related macular degeneration (AMD) and diabetic retinopathy (DR), which leads to vision loss [2,3]. Breakdown of BRB is an important pathophysiological parameter in macular edema in AMD and DR [4,5]. Several inflammatory factors, comprising vascular endothelial growth factor (VEGF) and intercellular adhesion molecule-1 (ICAM-1), are involved in the breakdown of the BRB [6]. Furthermore, leukostasis or leukocyte adherence to the retinal vasculature has been confirmed to be raised in the retina of both AMD and DR animal models [7]. Likewise, macular edema and increased vascular permeability are the outcomes of the subsequent damage to the capillary endothelium [7,8].

AMD is a main cause of irreversible blindness among individuals aged over 50 years, affecting approximately 30% of those over 80 years [9]. According to projections, there could be 288 million AMD patients by 2040 [10,11]. AMD is predicted to reach 113 million cases by 2040, with most patients currently found in areas with a higher proportion of aging populations. However, in the future, the demographics may change to Asian countries [12].

AMD is a long-term, progressive neurodegenerative condition and a major cause of permanent vision loss and blindness globally [13]. AMD can be defined as a chronic, complex disease that arises because of multiple genetic variants, such as the Complement factor H-associated gene, environmental factors like exposure to cigarette smoke and UV radiation, and lifestyle choices, for instance, smoking [11,14]. The AMD can be classified based on clinical characteristics into dry (non-vascular or early stage) AMD and wet AMD (neovascular or late stage), characterized by neovascularization in the macula [15]. The dry AMD accounts for 90% of cases, and it is typically managed by advising patients to avoid smoking, maintain a healthy lifestyle, and take antioxidant supplements.

In the case of wet AMD, current clinical therapies are based on targeting vascular endothelial growth factors (VEGF) with recently approved anti-VEGF antibodies [16]. Anti-VEGF medications, such as bevacizumab, aflibercept, and ranibizumab, are injected into the eye to inhibit VEGF activity and reduce the development of unusual blood vessels [17,18]. The administration of these antibodies to the target site is a challenging task due to poor permeability after topical administration; therefore, multiple intravitreal injections are used, which can lead to patient noncompliance and increase the risk of intraocular infections [19,20].

ALA (ω-3) and LA (ω-6) are essential fatty acids (EFAs) [21]. The systematic reviews, clinical studies, and meta-analyses of randomized clinical trials exhibit that dietary consumption of ω-3 FAs relieves symptoms of dry eye by reducing inflammation via the synthesis of anti-inflammatory prostaglandin E3, which decreases the production of pro-inflammatory mediators [22,23]. The focus on EFAs has also now shifted from dietary supplementation to topical application, delivering EFAs directly to the ocular surface. Topical applications of EFAs have been shown to reduce inflammation at the ocular surface in animal models [24,25] and human clinical trials [26], leading to prospects for the commercialization of EFAs, including eye drops for treating dry eye.

Microemulsion (ME) is a thermodynamically stable and spontaneously formed submicron drug delivery carrier comprising an appropriate ratio of oil, surfactant, cosurfactant, and aqueous phase. It is suitable for poorly soluble drugs and provides physical stability and safety to the drug, as reported previously [27,28]. As compared to conventional eye drops, ME has various advantages, including sustained drug action, less frequent instillation, enhanced ocular retention, good permeation into the cornea, improved bioavailability of the ocular drug, and better patient compliance [29,30,31].

Hence, this study aimed to formulate an ocular delivery system of ME for topical applications and optimize the formulations by designing pseudo-ternary phase diagrams. Then, the physicochemical properties of drug-loaded ME were characterized, and the in vitro drug release studies were examined. The HET-CAM study was conducted to evaluate the irritation potential and anti-angiogenesis activity of blank ME and drug-loaded ME. Further, RAW 264.7 cells were used to check cell cytotoxicity along with the LPS-induced anti-inflammatory activity of optimized ME.

## 2. Materials and Methods

### 2.1. Chemicals, Antibodies, and Cells

The reagents used in the invention are as follows. Penicillin (100 units/mL)/streptomycin (100 g/mL) and FBS (fetal bovine serum), 0.25% trypsin-EDTA, Cell Titer 96 Aqueous one solution cell proliferation assay kit (Prommega, Madison, WI, USA), LPS (lipopolysaccharide) (Sigma Aldrich, St. Louis, MO, USA), Halt Protease Inhibitor Cocktail, EDTA-Free (Thermo Scientific, Waltham, MA, USA), M-PER^®^ Mammalian Protein Extraction Reagent (Thermo Scientific Waltham, MA, USA), Western Enhanced Buffer (NEOSCIENCE, Seoul, Republic of Korea), Super Signal^®^ West Pico Chemical Substrate (Thermo Scientific Waltham, MA, USA), PVDF membrane (Sigma Aldrich, St. Louis, MO, USA), phosphate-buffered saline (PBS), sodium hydroxide, hydrochloric acid, and other reagents were purchased from Sigma Aldrich. The cell line used in the study was mouse monocyte RAW 264.7 (KTCC No. 40071) purchased from Korea Cellular Bank (KTCC, Seoul, Republic of Korea). Emulsion manufacturing materials include alpha-linolenic acid (CAS No. 463-40-1), Trancutol HP (Diethylene glycol monoethyl ether, CAS No. 111-90-0), Cremorphor EL40 (PEG-35 castor) Drugs such as oil, CAS No. 61791-12-6), Span 80 (Sorbitan monooleate, CAS No. 1338-43-8) and Axitinib (CAS No. 319460-85-0) was purchased from Merck Co, Darmstadt, Germany. Sirolimus (CAS No. 53123-88-9) and Fenofibrate (CAS No. 49562-28-9) were purchased from Hangzhou Sartort Biopharma Co., Ltd., Hangzhou, Zhejiang, China. For Western blot analysis related to inflammation in response to substances, the primary antibodies were anti-COX-2, anti-IL-1β polyclonal antibody (GeneTex, Irvine, CA, USA), and anti-β-Actin antibody (Santa Cruz Biotechnology, Dallas, TX, USA), and the secondary antibody was Goat Anti-Rabbit IgG antibody (HRP) (GeneTex, Irvine, CA, USA).

### 2.2. Construction of Pseudo-Ternary Diagram(s)

Microemulsion is a homogeneous system that combines the optimum ratios of oil, surfactant, cosurfactant, and aqueous phase. The determination of the relative ratio can be performed by constructing pseudo-ternary phase diagrams using the titration method. Pseudo-ternary phase diagrams were constructed using water titration at ambient room temperature. The weight ratios of the surfactant mixture (S_mix_) were set at 1:1:1, 1:1:0.5, and 1:1:1.5. These ratios were determined by titrating the oil and S_mix_ in a 1:1 ratio with an aqueous phase. The weight ratios of oil to mixed surfactants were kept at 1:9, 2:8, 3:7, 4:6, 5:5, 6:4, 7:3, 8:2, and 9:1 to construct the ternary phase diagram. The oil and S_mix_ were mixed homogeneously, and the aqueous phase was added dropwise under continuous mixing using a vortex mixture at room temperature until a transparent/translucent system was produced. The point was noted when the solution mixture turned hazy or turbid. A pseudo-ternary phase diagram was drawn by calculating the concentrations of these components.

### 2.3. Preparation of Blank and Drug-Loaded Formulations

**Figure 1:**
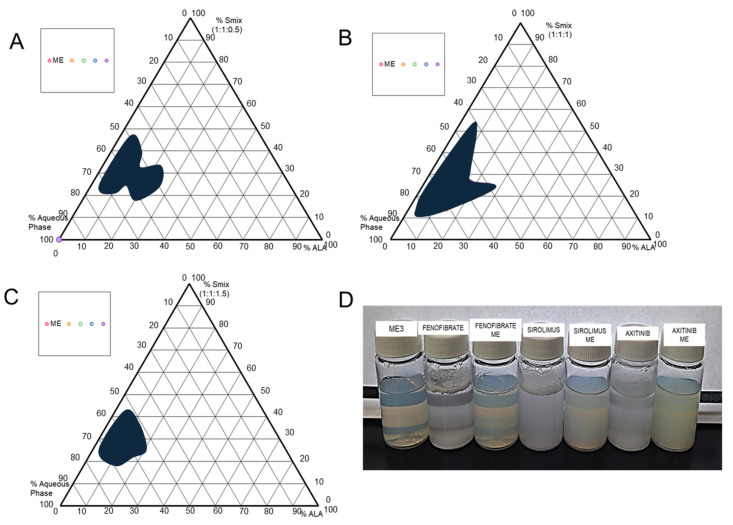
Pseudo-ternary phase diagrams of different ratios of alpha-linolenic acid with surfactant mixture (S_mix_). S_mix_: Cremophor RH 40: Span 80: Transcutol P, respectively (**A**) S_mix_ 1:1:0.5, (**B**) S_mix_ 1:1:1, (**C**) S_mix_ 1:1:1.5, and (**D**) optimized ME3, pure drug solution in water with ME.

Six ME formulations (three blank and three drug-loaded) were prepared. The required drug quantity was added to the various proportions of oil, surfactant, and cosurfactant selected from pseudo-ternary phase diagrams (Figure 1 and Table 1). The drug was dissolved in the mixture of oil and S_mix_ by constant stirring on a magnetic stirrer; then, the required volume of the aqueous phase was added and mixed well to obtain the homogeneous ME. Similarly, without a drug, ME was also prepared.

### 2.4. Determination of Drug Content and pH Measurement

The drug content of the prepared ME was determined by diluting it with methanol. Briefly, 0.2 mL of each prepared ME was diluted up to 10 mL of methanol separately in a calibrated volumetric flask, and an aliquot of this solution was suitably further diluted with methanol and analyzed by a UV spectrophotometer at respective wavelengths. The drug content was determined in triplicate.

To confirm that the ME formulation met the requirements for eye drops, the pH needed to be tested. The pH value of the ME formulation was measured by a pH meter (Orion Star A211, Thermo Scientific, Waltham, MA, USA). All ME samples were tested in triplicate.

### 2.5. Globule Size and Polydispersity Index (PDI)

The average globule size and PDI of prepared formulations were analyzed by photon correlation spectroscopy. Measurements were made using Zetasizer 1000 HS (Zen 3600, Malvern Instruments, Worcestershire, UK), wherein light scattering was monitored at 25 °C at an angle of 90°.

### 2.6. Surface Morphology and Structure

Prepared formulation samples were subjected to morphological and structural analysis using transmission electron microscopy (TEM, H7500, Tokyo, Japan) machine running at 100 kV with a point-to-point resolution. Copper electron microscopy grids were mounted onto a supporting plate to prepare samples for negative staining. After that, samples were progressively dropped onto the copper grid and allowed to air dry for around fifteen minutes. The film was then treated with a drop of 2% phosphotungstic acid and allowed to dry for an additional ten minutes before being examined under a TEM.

### 2.7. Thermodynamic Stability

Various thermodynamic stability tests, like heating–cooling cycle, centrifugation, and freeze–thaw cycle, were performed on selected ME [32]. ME Formulations that passed the above stress tests were additionally subjected to a dispersibility test.

### 2.8. Dispersibility Test

This test evaluates the efficiency of self-emulsification for drug-loaded ME. Briefly, 1 mL of each ME formulation was added to 500 mL of water at 37 ± 0.5 °C, and the in vitro emulsification rates as well as the appearance of microemulsions were graded visually, as reported previously [28].

### 2.9. In Vitro Drug Release

A volume of 1.0 mL of the formulation was enclosed in a dialysis bag (cellulose membrane, MW cut-off 12400) and incubated in 250 mL release medium at 37 ± 0.5 °C under mild agitation in a water bath. Simulated tear fluid was employed as the release medium. At predetermined time intervals, 1.0 mL of the sample was withdrawn from the release medium and analyzed by using UV spectrophotometry. After sampling, 1.0 mL of thermoregulated fresh medium was added to the release medium. The experiment was repeated three times.

### 2.10. Cytotoxicity Test of ME

MTS [3-(4,5-dimethylthiazol-2-yl)-5-(3-carboxymethoxyphenyl)-2-(4-sulfophenyl)-2H-tetrazolium, inner salt] assay was performed to measure the toxicity or cell proliferation effects of the blank ME and drug-loaded ME on cells at concentrations of 0.001, 0.01, 0.1, and 1 μg/mL. Briefly, cultured RAW 264.7 cells were distributed uniformly. All cells were cultured under dark conditions with 5% CO_2_ and 37 °C. The Cell Titer 96^Ⓡ^AQueous One Solution Cell Proliferation Assay Kit (Promega, Madison, WI, USA) was used to determine the effect on cell proliferation by treatment at different sample concentrations. After processing each test formulation, all medium was removed, and 20 μL MTS solution was added to 100 μL of the new DMEM. Afterward, the cell reaction mixture was kept for 3 h in the dark at 5% CO_2_ and 37 °C, and the absorbance was measured at 490 nm using an ELISA measuring device. A culture medium without any test sample treatment was used as a control. The final cell viability was calculated.

### 2.11. Evaluation of Protein Expression Related to the Inflammatory Response

The anti-inflammatory efficacy of each formulation was tested at concentrations of 0.001, 0.01, 0.1, and 1 μg/mL. The developed blank ME, Quercetin (QU), Sirolimus, and Sirolimus ME were added with macrophages (RAW 264.7 cells), which induced inflammation with LPS, and then cell proliferation and cytotoxicity were examined through MTS analysis. Also, the anti-inflammatory effect of each formulation was analyzed using the RAW 264.7 cell line treated with the inflammation-inducing substance LPS. Cell lines were cultured in DMEM (Dulbecco’s Modified Eagle medium) containing 10% fetal bovine serum (FBS) (HyClone, Logan, UT, USA) and penicillin (100 units/mL)/streptomycin (100 μg/mL) (HyClone, Logan, UT, USA).

Furthermore, the cells were cultured in Eagle’s Medium at 37 °C in a CO_2_ cell incubator (NU-4750G, NuAire, Plymouth, MN, USA) with 5% CO_2_. The inflammatory response to each formulation was investigated to evaluate the degree of regulation of expression of inflammation-related indicator proteins COX-2 and IL1-β. The expression level of β-actin was analyzed as a control protein to quantify protein expression in cells treated with each formulation. This was carried out utilizing the Western blot method with antibodies. The expression level of indicator proteins was analyzed using ImageJ^®^ analyzer (National Institutes of Health, Bethesda, MD, USA).

### 2.12. Irritation and Anti-Angiogenesis Study Using Hen’s Egg Test–Chorioallantoic Membrane (HET-CAM)

The irritation test is performed to mimic the non-irritancy of the formulation on the vascular tissue of HET-CAM. The fertilized hen’s egg was procured from a local poultry farm and steadily placed inside the incubator (set at 37 °C, humidity 50–60%) after wiping with 70% alcohol to prohibit any contamination. Eggs were rotated every 12 h to allow the formation of a membrane at the circumference.

On day 3, egg albumin (2 mL) was removed carefully from the cuspid pole of the egg using a sterile needle while maintaining a sterile condition. The hole was sealed using parafilm (previously sterilized with 70% *v*/*v* alcohol) with the help of a heated spatula (pre-sterilized). Afterward, the eggs were placed with the blunt pole containing the air space facing upwards and incubated for 2 days without further rotation. The eggs were kept this way so that the development of the chorioallantoic membrane (CAM) takes place away from the apical shell. On the 10th day, with the help of forceps, a window of 2 × 2 cm was carefully made on the eggshell, through which the test formulation with appropriate dilution was applied directly onto the CAM surface and checked for the reaction. The reaction of blood vessels to the formulation was observed, and morphological changes were recorded for 5 min following the start of the exposure. The time taken for vascular damage to the membrane was examined and recorded.

In this experiment, NaOH solution was utilized as a positive control, and 0.9% NaCl as a negative control. Blank ME, Axitinib ME, Sirolimus ME, and Fenofibrate ME were used as test formulations. These eggs were photographed at the starting and 5 min time points to observe for any changes. The one that would not harm the normal vasculature present in egg embryos would be referred to as non-irritant.

Furthermore, the same treatment was left for 24 h, and CAM was observed for vascular damage or changes to check the anti-angiogenesis effect of the developed ME.

### 2.13. Statistical Analysis

All experiments were repeated three times, and statistical analysis was expressed as mean ± standard deviation, using Student’s *t*-test method, and statistically significant when the *p*-value was found to be less than 0.05.

## 3. Results

### 3.1. Construction of Pseudo-Ternary Phase Diagrams

The pseudo-ternary phase diagrams of various S_mix_ ratios of 1:1:0.5, 1:1:1, and 1:1:1.5 were constructed using ALA as the oil phase, Cremophor RH40, Span 80, and Transcutol HP as the surfactant mixture (S_mix_). S_mix_ ratios 1:1:0.5 and 1:1:1.5 showed less microemulsion region (Figure 1A,C) than S_mix_ ratio 1:1:1 (Figure 1B). That could be due to the lower solubilization capacity of oil in the ratio of S_mix_.

Furthermore, analysis of the pseudo-ternary phase diagram (Figure 1B) confirmed that the optimal compositional ratios for the components were found in the range of 10–50% for the S_mix_, 5–30% for ALA, and 40–90% for the aqueous phase. Hence, the pseudo-ternary phase graph constructed with a S_mix_ ratio of 1:1:1 can provide a wide range for the selection of microemulsion compositions.

### 3.2. Preparation of Microemulsions

ME formulation components were selected from the pseudo-ternary phase diagram, which has the largest region for a transparent and one-phase, low-viscosity ME system (Figure 1). ALA was utilized as an oil phase, and purified water as the aqueous phase. The S_mix_ (1:1:1) showed the highest microemulsion region, as observed in Figure 1B. This could be due to the good emulsification of the oil phase with the selected S_mix_ ratio of Cremophor RH 40, Span 80, and Transcutol P (1:1:1).

Table 1 presents the compositional ratios of different ME. Three blank microemulsions (ME1, ME2, and ME3) were prepared by changing the oil phase concentration as shown in Table 1. Based on the particle size, the most suitable ratio of S_mix_, oil, and the aqueous phase was found for ME3, which contains 20% S_mix_, 6.67% oil phase, and 73.33% aqueous phase (Table 1). Furthermore, to evaluate the drug loading capacity of the ME carrier, the dispersion of colloidal particles was selected based on the lowest particle size and polydispersity index (PDI) to load the drug. Hence, drug-loaded ME formulations containing Fenofibrate, Sirolimus, and Axitinib were developed and evaluated for particle size and PDI, as shown in Table 1. Blank and drug-loaded ME are shown in Figure 1D against the pure drug in double-distilled water.

### 3.3. Characterization of MEs

**Figure 2:**
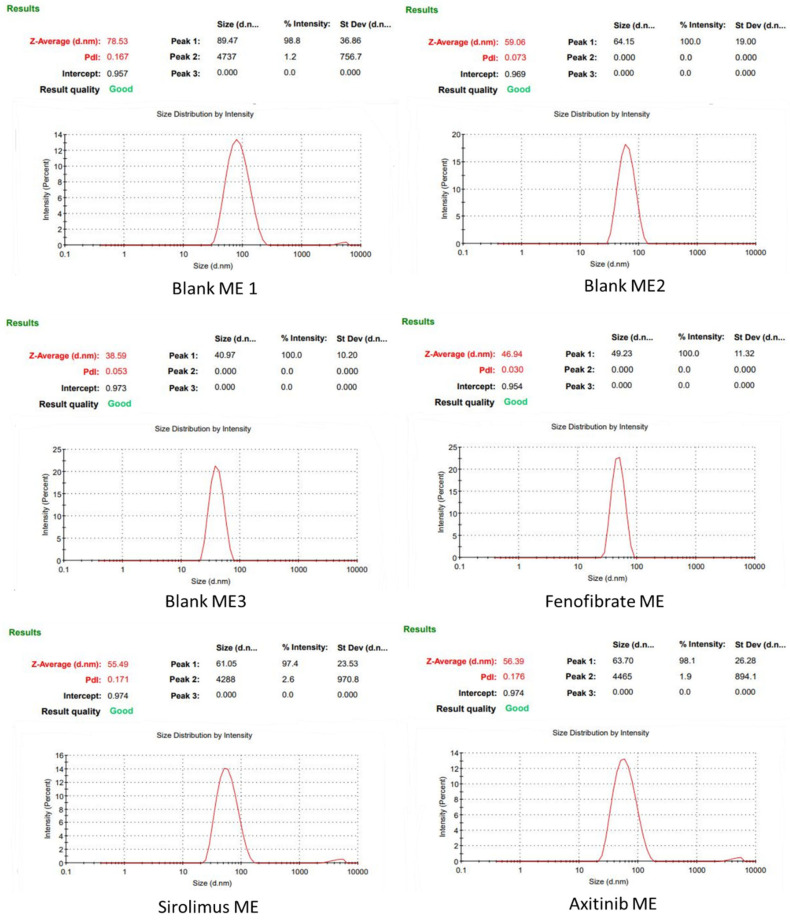
Results of the measured globule sizes of individual ME formulations.

The globule size of the ME formulations was measured using a Zetasizer Nano ZS (Zen 3600, Malvern Instruments, Worcestershire, UK), and results are shown in Table 1 and Figure 2. Among the prepared blank ME, the final formulation ME3, composed of 20% S_mix_, 6.67% ALA, and 73.33% aqueous phase, formed a homogeneous ME with the smallest average particle size of 38.59 nm (Table 1). ME3 displayed a low polydispersity index (PDI) of 0.053, indicating that the measured droplet particles were highly homogeneous. The droplet sizes of drug-loaded ME formulations were confirmed to be approximately in the range of 47–56 nm (Table 1 and Figure 2). The Fenofibrate ME exhibited an average diameter of approximately 47 nm and showed a highly homogeneous particle distribution (PDI = 0.03). Additionally, the Sirolimus and Axitinib formulations yielded homogeneous particles with an average globule size of approximately 55 nm (Table 1).

### 3.4. Drug Content and pH

The drug content in all the drug-loaded ME was observed to be more than 90%, which varied in the range of 92–98% (Table 2). Therefore, the results indicated the uniform distribution of the drug in the ME formulations.

The pH values of the prepared drug-loaded ME formulations varied from 6.19 to 6.68 (Table 2).

### 3.5. Thermodynamic Stability and Dispersibility Test

**Figure 3:**
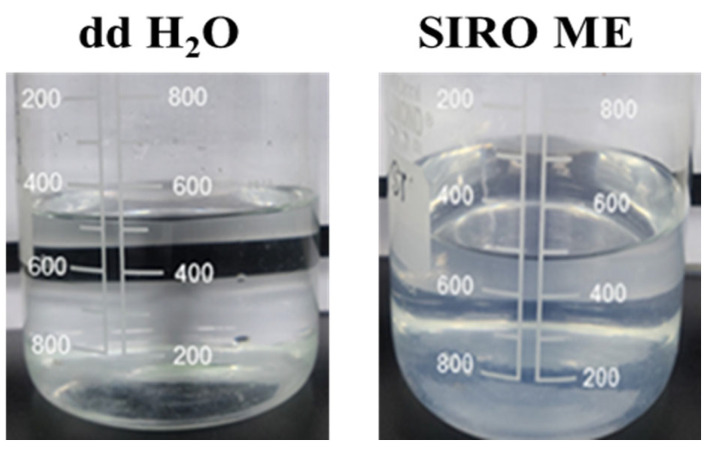
Dispersibility test results of the Sirolimus-loaded microemulsion against double-distilled water.

ME formulations, which remained stable after exposure to thermodynamic variations, were taken for dispersibility tests (based on emulsification rate and appearance). The results of the dispersibility test are shown in Figure 3. The ME formulations were graded via the dispersibility test as Grade A (rapid and clear), B (rapid and bluish white), C (moderate and milky white), D (gradual and grayish white), and E (gradual with the presence of large oil globules) [28]. Grades A and B can be selected for further study, as these formulations will remain stable when dispersed at targeted sites. For Grades A and B, there was no drug precipitation upon dilution, demonstrating that the ME formed could keep the drug solubilized. Grades C (moderate and milky white), D (gradual and grayish white), and E (gradual with the presence of large oil globules) were discarded due to the possibility of phase separation on dilution, which could lead to precipitation of poorly soluble drug. The Sirolimus and Fenofibrate-loaded ME showed the Grade A type ME, while Axitinib-loaded ME showed Grade B type ME (Table 2 and Figure 3). This could be because of the difference in drug solubility in the ME.

### 3.6. Structure and Morphology by Transmission Electron Microscopy (TEM)

**Figure 4:**
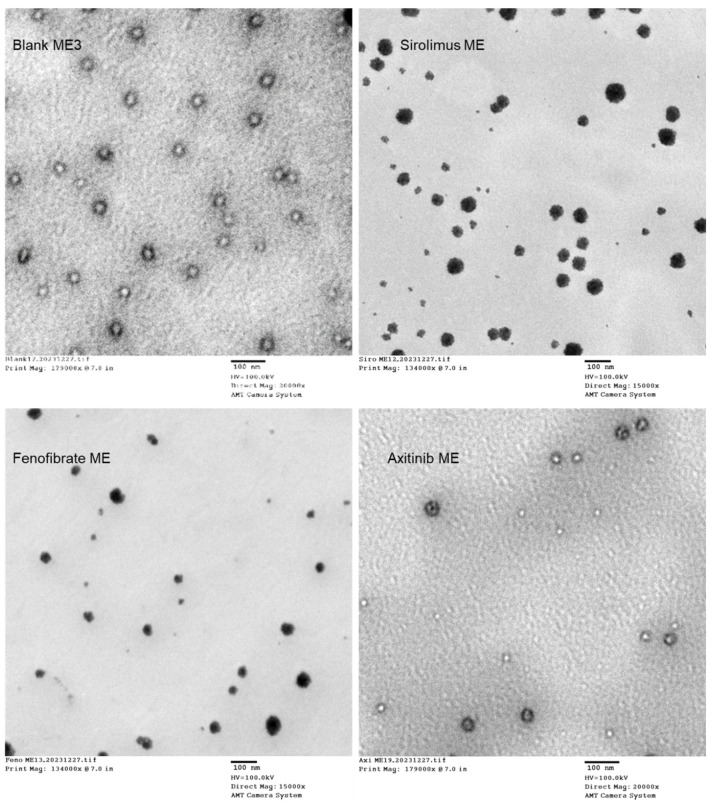
Appearance of ME observed through transmission electron microscopy (TEM).

TEM was used to examine the structures and morphologies of the prepared MEs. The results confirmed that ME exhibited globule or droplet formation in the nano range (Figure 4). The interphase of oil, Cremophor RH 40, Transcutol P, and Span 80 displayed a denser region, indicating film formation, which prevents the globules from coalescing.

### 3.7. In Vitro Drug Release from the Developed Formulation

**Figure 5:**
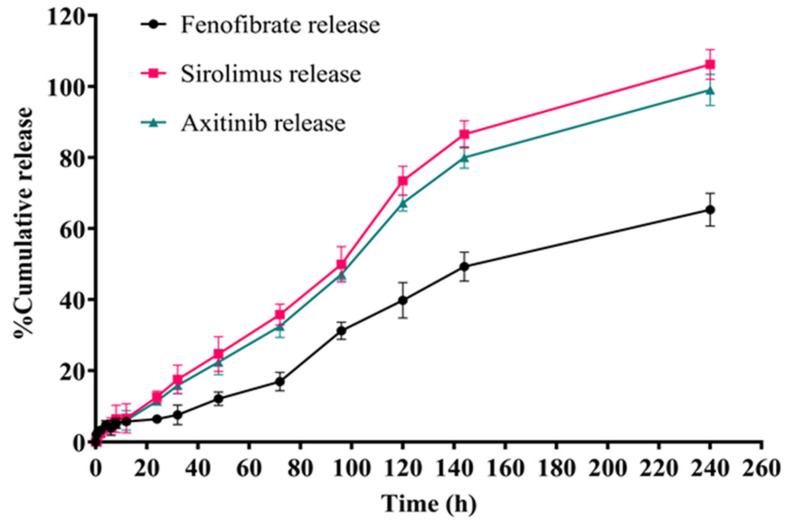
In vitro drug release graphs from prepared drug-loaded ME formulations.

The drug release studies were conducted for the newly developed ME formulations containing encapsulated drugs (Figure 5), i.e., Axitinib ME, Fenofibrate ME, and Sirolimus ME. The results of the release studies for the MEs indicated that the cumulative release increased over time for all ME formulations (Figure 5). Remarkably, the in vitro drug release profile revealed that Sirolimus exhibited the highest release, followed by Fenofibrate, while Axitinib demonstrated the lowest release amount (Figure 5).

In the present study, ME formulations, each prepared with Sirolimus, Fenofibrate, and Axitinib, showed the respective drug release in STF medium (pH 7.4): 12.671 ± 1.063%, 6.433 ± 1.263%, and 11.492 ± 1.062% at the end of 24 h, while 106.178 ± 4.203%, 65.315 ± 4.624%, and 98.993 ± 4.427% at the end of 240 h, respectively. The release pattern confirmed the sustained release of drug(s) from the ME (Figure 5).

### 3.8. Evaluation of Irritation and Vascular Changes (Anti-Angiogenesis) via HET-CAM (Hen’s Egg Test—Chorioallantoic Membrane)

**Figure 6:**
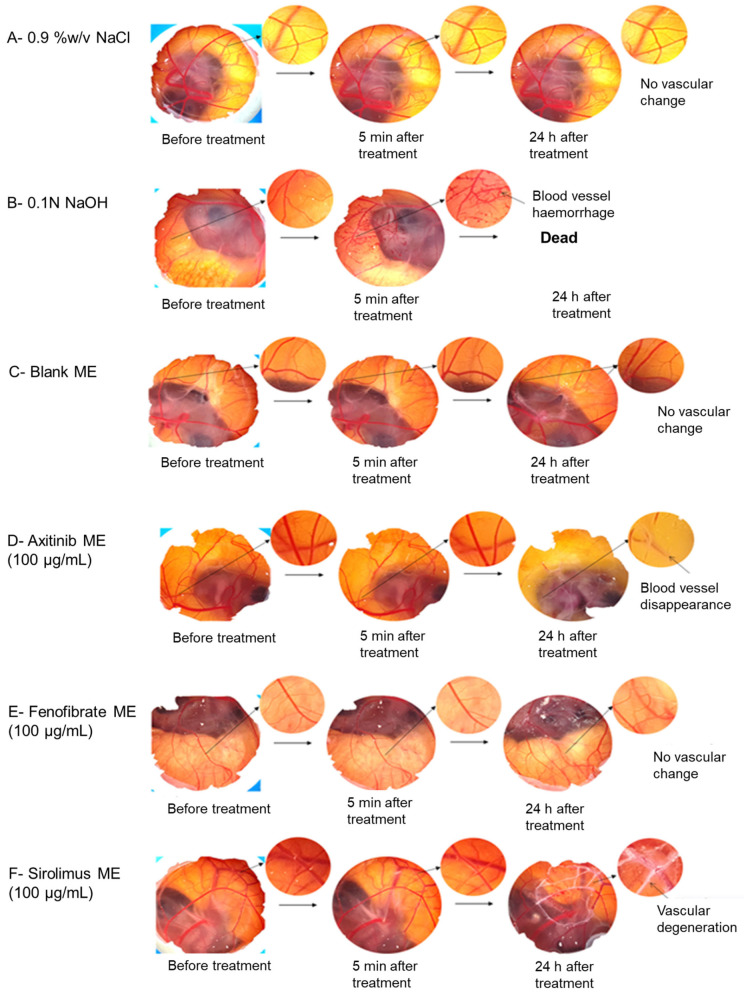
HET-CAM (Hen’s Egg Test—Chorioallantoic Membrane) assay was conducted to evaluate each microemulsion’s effects on vascular alterations. HET-CAM images following treatment with (**A**) Sodium chloride (0.9% NaCl), (**B**) Sodium hydroxide (0.1 N NaOH), (**C**) Blank ME, (**D**) Axitinib ME, (**E**) Fenofibrate ME, and (**F**) Sirolimus ME.

To assess the irritation potential and vascular changes induced by the developed ME, the HET-CAM (Hen’s Egg Test—Chorioallantoic Membrane) method, which serves as an alternative to animal testing, was employed (Figure 6). Following the application of the positive control (0.1 N NaOH), negative control (0.9% NaCl), blank ME, and drug loaded ME onto the blood vessels formed in the chorioallantoic membrane of fertilized chicken eggs, observations of the vascular changes were conducted after a predetermined time interval (0 min, 5 min, and 24 h). The results, presented in Figure 6, depict the vascular alterations after post-application of the Fenofibrate ME and Sirolimus ME onto the chorioallantoic membrane in comparison with positive, negative, and standard Axitinib ME.

At the end of 5 min, the results of the HET-CAM test indicate that the developed blank and drug-loaded ME did not cause any damage to the blood vessels and exhibited no sensitizing effects, hence demonstrating their non-irritant properties as compared to the positive control (Figure 6). The mean score for 0.9% NaCl (Figure 6A), Blank ME (Figure 6C), Axitinib ME (Figure 6D), Fenofibrate ME (Figure 6E), and Sirolimus ME (Figure 6F) was observed 0 after 5 min of HET-CAM treatment with no damage to the blood vessels (normal network of capillary architecture); thus, the developed formulations were categorized as non-irritant. In addition, the 0.1 N NaOH-treated group (Figure 6B) displayed a mean score of 16.67 (Table 3) with substantial rupture of the blood vessels and thus was categorized as a positive irritant.

Furthermore, the anti-angiogenic activity of the prepared ME (equivalent to a 100 μg/mL dose) was compared to that of 0.9% NaCl. At the end of 24 h, the Sirolimus-loaded ME showed the inhibition of neovascularization or sprouting of blood vessels as compared to the Fenofibrate-loaded ME (Figure 6E,F).

**Figure 7:**
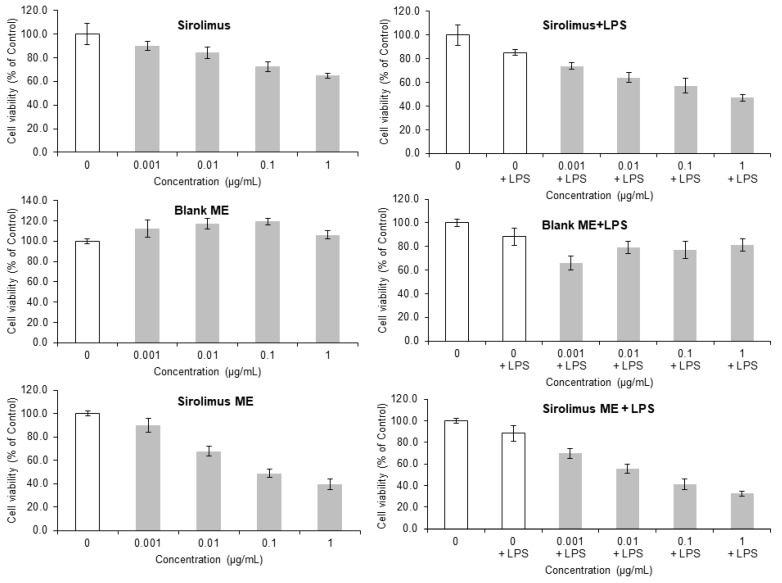
Concentration-dependent cell viability results of pure Sirolimus, blank ME, and Sirolimus ME.

**Figure 8:**
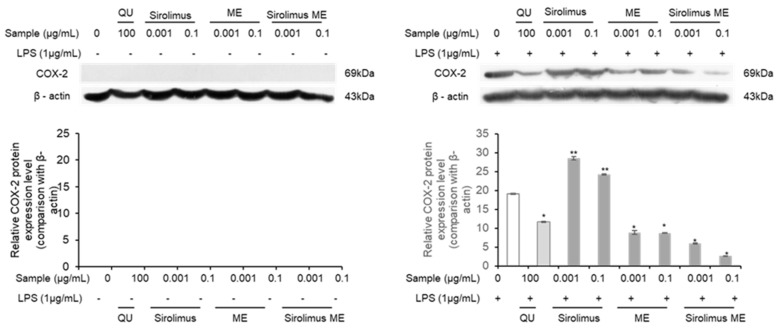
Western blot analysis of the expression levels of COX-2 proteins as inflammation-related biomarkers in LPS-induced macrophage RAW 264.7 cell line following treatment of Sirolimus, blank ME, and Sirolimus ME with respect to control and quercetin. (** means no significant difference as compared to control at *p* < 0.05, * means significant difference at *p* < 0.05 as compared to control).

Our results of Sirolimus ME also exhibited the reduction in blood vessel sprouting in the HET-CAM test in anti-angiogenesis activity (Figure 6F) as compared to 0.9% NaCl and Fenofibrate ME. Because the localized damages of HET-CAM blood vessels in the treated region were more pronounced in the case of Sirolimus ME against the normal capillary network with 0.9% NaCl treatment, as shown in Figure 6 (Figure 6A,F). This indicated that the enhanced anti-angiogenesis potential of Sirolimus ME, which can be beneficial in the treatment of wet AMD. Additionally, based on HET-CAM results, blank ME, and Sirolimus ME were tested for cytotoxicity test and COX-2 protein expression assay in RAW 264.7 cells (Figure 7 and Figure 8).

### 3.9. Cell Cytotoxicity Assay and Evaluation of Inflammatory Response-Related Protein Expression

The MTS assay is used to determine the degree of toxicity and also measures the cell viability of a new product or new substances. The cell cytotoxicity results are shown in Figure 7. The cytotoxicity and effects on cell proliferation of the pure drug, blank ME, and the drug-loaded ME were investigated on RAW 264.7 cells without LPS and with LPS treatment. It is known that LPS acts as a pro-inflammatory factor, activating monocytes and converting them into macrophages to respond to inflammatory stimuli [33].

Pure Sirolimus and Sirolimus ME revealed the concentration-dependent cell cytotoxicity in RAW 264.7 cells without LPS and with LPS treatment. It was observed that the ME exhibited no significant cytotoxicity at concentrations of 0.001, 0.01, 0.1, and 1 μg/mL in the absence of LPS, demonstrating over 100% cell viability compared to the untreated control group (Figure 7). But in the presence of LPS, at 0.001 μg/mL, the ME showed about 60% cell viability, while at concentrations of 0.01, 0.1, and 1 μg/mL, it exhibited around 80% cell viability. The cell cytotoxicity effect may be due to inflammation caused by LPS. Since the ME contained ALA, the ME may have some anti-inflammatory effect, as reported previously [34]. Thus, it can be concluded that the newly developed ME itself is non-toxic to cells.

Furthermore, cell cytotoxicity (IC50) was observed in Sirolimus without and with LPS, 3.62 μg/mL and 2.80 μg/mL, while the Sirolimus ME showed IC50 1.88 μg/mL and 1.85 μg/mL, respectively, in a dose-dependent manner. Moreover, the Sirolimus ME treatment at concentrations of 0.01, 0.1, and 1 μg/mL resulted in cell proliferation rates of 67.8%, 45.9%, and 28.4%, respectively (Figure 7). Additionally, in LPS-induced inflammatory RAW 264.7 macrophage cells treated with Sirolimus ME, a gradual decrease in cell proliferation rates was also observed (Figure 7).

The expression of various pro-inflammatory cytokines and enzymes, including iNOS, COX-2, TNF-α, IL-6, and NF-kB, is a recognized biomarker of cellular inflammation responses [33].

Further, the expression changes in the key inflammatory factor COX-2 were assessed following the treatment of LPS-induced RAW 264.7 cells with the newly developed ME and Sirolimus MEs using Western blot analysis. Cyclooxygenase (COX) is an enzyme that catalyzes the conversion of arachidonic acid into prostaglandins (PGs) and thromboxanes. Hence, COX-2 is recognized as an inflammatory factor that is upregulated in tissues during inflammation.

Additionally, quercetin, known for its potent anti-inflammatory effects, was included as a positive control in the experiment. We have also observed quercetin and quercetin nanosuspension for anti-inflammatory activity in LPS-induced RAW 264.7 macrophage cells in our previous research [33,35].

In contrast, when the newly developed ME, Sirolimus, Sirolimus ME, and quercetin were tested in RAW 264.7 macrophage cells with lipopolysaccharide (LPS) induced inflammation, the expression of COX-2 was observed (Figure 8). It was observed that the treatment with the positive control quercetin (100 μg/mL) suppressed COX-2 expression. Conversely, treatment with Sirolimus at concentrations of 0.001 μg/mL and 0.1 μg/mL led to an increase in COX-2 expression compared to the control cells (Figure 8). However, treatment with the newly developed ME at concentrations of 0.001 μg/mL and 0.1 μg/mL resulted in suppression of COX-2 (Figure 8). Similarly, treatment with the Sirolimus ME at the same concentrations also significantly (*p* < 0.05) inhibited the expression of COX-2.

## 4. Discussion

AMD is a progressive retinal disease that mainly damages central vision by destroying the macula (light-sensitive region of the retina), especially in older people [36]. It is two types: dry (atrophic) AMD, in which thinning of the macula and buildup of drusen occurs, and wet (neovascular) AMD, which is recognized by abnormal blood vessels growing underneath the retina that can leak fluid or blood. In dry AMD, there is no cure, but certain high-dose antioxidant and mineral supplements can slow progression in intermediate or advanced cases, along with lifestyle changes such as non-smoking, diet, and blood pressure control [37]. For wet AMD, the mainstay is intravitreal injections of anti-VEGF (vascular endothelial growth factor) agents to reduce neovascularization and leakage, supplemented in some cases by photodynamic therapy or laser treatment. Problems and limitations remain: for dry AMD, there has long been no effective therapy to reverse damage or restore lost vision, and even the newer treatments only slow degeneration. For wet AMD, anti-VEGF treatments require frequent injections (often monthly or bi-monthly), which can burden patients, and a subset of patients respond poorly or develop resistance over time. Other treatments, like surgery, have generally failed to produce reliable improvements for many lesion types. Because of complications or side effects of intravitreal injections, we have tried to develop a topical delivery system based on ME to reduce the side effects and increase patient compliance. MEs are defined as isotropic and thermodynamically stable liquids formed by mixing oil, surfactant, and water [38]. In this study, MEs were developed by constructing the pseudo-ternary phase diagram using the titration method [39].

The pseudo-ternary phase diagrams comprising ALA, Cremophor RH40, Span 80, Transcutol HP, and water displayed a region of microemulsion formation (shaded area) at room temperature, as shown in Figure 1. Pseudo-ternary phase diagrams are constructed to study the association between the phase behavior and the composition of the MEs. Phase diagrams also help to decide the concentration series of components for the formation of MEs. To minimize the potential toxicity of high surfactant content and to avoid gel formation due to the high content of Cremophor RH40 and increase the drug solubility, cosurfactants were selected. The results of selected compositions indicated that a microemulsion formed with the surfactant mixture (S_mix_) at a ratio of 1:1:1 (Figure 1B) was observed as more transparent compared to those with S_mix_ ratios of 1:1:0.5 and 1:1:1.5 (Figure 1A,C). Therefore, to minimize the potential toxicity associated with high surfactant content and to avoid gel formation due to the high concentration of Cremophor RH40, a surfactant mixture with a S_mix_ ratio of 1:1:1 was selected to obtain a clear homogeneous microemulsion [39]. Based on this, three blank ME were prepared as shown in Table 1. The optimized ME spontaneously formed as a fine oil-in-water (*o*/*w*) system when aqueous medium was added to the oil and S_mix_. This is because the free energy involved in making the ME is very low; hence, ME formation is spontaneous and thermodynamically stable [40]. Furthermore, since surfactants or surfactant mixtures make a layer around the droplets, they reduce the interfacial energy. Additionally, they also provide a mechanical barrier to prevent the coalescence of the droplets [41].

The globule size results indicated that the ME formulations were nano-sized and exhibited consistent homogeneity (PDI < 1) [42], as shown in Table 1 and Figure 2. It was observed that the particle size decreased as the concentration of the oil phase decreased (Table 1). This could be due to the good emulsification of the oil phase with a selected ratio of S_mix_, which makes the smaller globules of the ME formulation during development. The optimized ME3 comprised 20% S_mix_, 6.67% ALA, and 73.33% aqueous phase and showed a globule size of 38.59 nm, while the drug-loaded ME showed a globule size range of 47–56 nm. In addition, TEM results also confirmed the formation of ME globules in the nano range, and a dense interphase region was indicated in the film formation due to the presence of S_mix_ (Figure 4)

The drug-loaded ME formulations showed the drug loading range of 92–98% (Table 2). Therefore, the results indicated the uniform distribution of the drug in the ME formulations. Also, because of the good solubilization capacity of developed and selected ratios of components, the ME formulations load and distribute the drug uniformly into the ME globules.

The pH values of ME formulations varied from 6.19 to 6.68 (Table 2). Furthermore, it has been reported that the eye has a limited buffering capacity, as the ocular comfort ranges from pH of 6.6 to 7.8 for topical preparations [43]. But the pH of therapeutic substances applied as eye drops varies from 3.5 to 8.5, hence the pH values of the prepared ME could be considered to fall within the tolerable/acceptable range [44].

The thermodynamic stability test of blank ME and drug-loaded ME exhibited no phase separation, drug precipitation, or creaming. Based on the thermodynamic stability test, a dispersibility test was conducted for stable ME and graded as Grade A (rapid and clear), B (rapid and bluish white), C (moderate and milky white), D (gradual and grayish white), and E (gradual with the presence of large oil globules) [28]. Grades A and B can be selected for further study, as these formulations will remain stable when dispersed at targeted sites. The developed drug-loaded ME formulations displayed Grades A and B. Since this grade of ME showed no drug precipitation of drug upon dilution, indicating that the designed ME could keep the drug in a solubilized state. Hence, these ME may remain stable in storage and might not precipitate during administration for therapeutic purposes. Sirolimus ME was graded as Grade A, as shown in Figure 3.

Furthermore, the in vitro release studies of Axitinib ME, Fenofibrate ME, and Sirolimus ME results indicated the sustained release of drug(s) from the ME (Figure 5). It is known that the release pattern of a therapeutic moiety from a nanocarrier determines whether the target area will experience prolonged and continuous exposure at lower/safer concentrations (minimal side effects) for improved efficacy compared to the conventional treatment [45]. Hence, the developed ME can deliver a longer duration and a safe dose of the drug to the target site.

In addition, the HET-CAM test was employed to test the irritation potential and anti-angiogenesis activity of the developed drug-loaded ME (Figure 6). HET-CAM offers an appealing alternative to animal studies because of its affordability and ease of use, as using mammalian models for preclinical toxicity (irritation) evaluation of nanoformulations necessitates ethical clearance and a costly or time-consuming setup [46]. The developed blank and drug-loaded ME did not display any irritation symptoms as compared to the positive control at 5 min post-application (Figure 6). For anti-angiogenesis activity, the same experiment was continued for 24 h and observed for vascular changes.

Axitinib is a multireceptor tyrosine kinase inhibitor and a small molecule that works by blocking vascular endothelial growth factor (VEGF) receptors and platelet-derived growth factor (PDGF) receptors, responsible for developing neovascularization [47]. Hence, Axitinib significantly inhibited the blood vessels more efficiently in the HET-CAM test and acts as a stronger anti-angiogenesis drug than Sirolimus (Figure 6D). The Sirolimus ME exhibited a better anti-angiogenesis effect as compared to the negative control and Fenofibrate ME at 24 h for the HET-CAM anti-angiogenesis test, suggesting its potential application in the prevention or treatment of AMD. Similarly, chitosan- and poly(lactic-co-glycolic acid)-based Sirolimus nanoparticles were reported for their anti-angiogenesis effects [45]. Also, it has been observed that the activity of Sirolimus is due to VEGF inhibition and reduction in endothelial cell sprouting in retinal pigment epithelium [48]. Furthermore, Sirolimus (rapamycin) is a mammalian target of rapamycin (mTOR) inhibitor with immunosuppressive, antiproliferative, antiangiogenic, antifungal, anti-restenosis, and anti-inflammatory properties [49]. Hence, it can be concluded that, compared to standard Axitinib ME, Sirolimus ME has less anti-angiogenesis effect, but it has a more prominent anti-angiogenesis effect than Fenofibrate ME.

In addition, Sirolimus and Sirolimus ME showed the dose-dependent cell cytotoxicity as shown in Figure 7. At low doses, higher cytotoxicity may be due to increased intracellular uptake of Sirolimus and Sirolimus ME, also because of phagocytosis and avoidance of P-glycoprotein efflux. This could be advantageous, as Sirolimus is a substrate of P-glycoprotein (drug efflux transporters) that may impede its cellular accumulation [50]. Moreover, the sustained release of Sirolimus from the ME for a prolonged period could also be responsible for cell killing. Additionally, higher uptake of Sirolimus might directly increase the activity of Sirolimus and result in non-specific cell cytotoxicity, which could ultimately lead to dose reduction.

Moreover, the anti-inflammatory activity of blank ME, Sirolimus, and Sirolimus ME was examined in LPS-induced RAW 264.7 macrophage cells against standard quercetin for COX-2 inhibition. It was observed that blank ME and Sirolimus ME showed a significant (*p* < 0.05) downregulation of COX-2 as compared to the control, while pure Sirolimus did not show significant COX-2 downregulation as compared to the control and quercetin. This effect can be attributed to the anti-inflammatory properties of ALA, a component of the ME [51]. Hence, the present study suggests that the anti-inflammatory potential of ALA could be attributed to COX-2 inhibition.

Hence, it can be concluded that the developed ALA-based ME could be a potential anti-inflammatory and anti-angiogenesis drug delivery system for topical use in wet AMD.

## 5. Conclusions

We developed an ALA-based ME formulation for topical drug delivery and evaluated its anti-inflammatory and anti-angiogenesis activity to treat AMD. The drug-loaded ME was successfully formulated, which significantly enhanced drug solubility, and its physicochemical properties were found to be suitable for ocular application. In vitro release profiles confirmed that the drug loaded in ME was released in a very effective and controlled manner. Furthermore, ME formulation showed no irritation effect in the HET-CAM test against 0.1 N NaOH solution. The HET-CAM antiangiogenic study demonstrated that Sirolimus ME significantly suppressed angiogenesis relative to the Fenofibrate drug-loaded ME and control (0.9% NaCl). Sirolimus ME demonstrated concentration-dependent suppression of COX-2 expression in LPS-stimulated macrophage cells, showing anti-inflammatory activity. Overall, we suggest that Sirolimus ME could be developed as a safe and effective topical ocular drug delivery system for the treatment of AMD and can be a potential alternative to intravitreal injections.

## Figures and Tables

**Table 1:** Compositional ratios, particle size (nm), and homogeneity (PDI) of microemulsions.

Formulation	Drug (% *w*/*w*)	S_mix_ (% *w*/*w*)	Oil ALA (*w*/*w*)	Aqueous Phase (% *w*/*w*)	Particle Size (nm)	PDI
Blank ME 1	0.0	20.0	13.33	66.67	78.53	0.167
Blank ME 2	0.0	20.0	10.00	70.00	59.06	0.073
Blank ME 3	0.0	20.0	6.67	73.33	38.59	0.053
Axitinib ME	0.1	20.0	6.67	73.33	56.39	0.176
Fenofibrate ME	0.8	20.0	6.67	73.33	46.94	0.030
Sirolimus ME	0.3	20.0	6.67	73.33	55.49	0.171

ME: microemulsion, S_mix_: Cremophor RH 40, Transcutol P, Span 80 (1:1:1), Oil: Alpha-linolenic acid, PDI: polydispersity index, *w*/*w*: weight-by-weight ratio.

**Table 2:** Drug content and pH measurements.

Formulation	Drug Content	pH	Dispersibility Grade
Axitinib ME	92.46 ± 2.87	6.19 ± 0.43	B
Fenofibrate ME	96.88 ± 1.79	6.73 ± 0.26	A
Sirolimus ME	98.25 ± 1.83	6.68 ± 0.32	A

**Table 3:** HET-CAM irritation test score of various formulations and test controls.

Symptoms Score/ Formulation		Hemorrhage (A)			Hyperemia (B)			Coagulation (C)			Mean Scores for 5 min A + B + C/3
		Time (min)			Time (min)			Time (min)			
	Egg	0.5	2	5	0.5	2	5	0.5	2	5	
0.9% NaCl	Egg 1	0	0	0	0	0	0	0	0	0	0
	Egg 2	0	0	0	0	0	0	0	0	0	
	Egg 3	0	0	0	0	0	0	0	0	0	
0.1 M NaOH	Egg 1	1	2	4	0	1	1	1	2	4	16.67
	Egg 2	1	2	4	0	1	1	1	3	3	
	Egg 3	2	2	5	0	1	1	1	3	3	
Blank ME	Egg 1	0	0	0	0	0	0	0	0	0	0
	Egg 2	0	0	0	0	0	0	0	0	0	
	Egg 3	0	0	0	0	0	0	0	0	0	
Axitinib ME	Egg 1	0	0	0	0	0	0	0	0	0	0
	Egg 2	0	0	0	0	0	0	0	0	0	
	Egg 3	0	0	0	0	0	0	0	0	0	
Fenofibrate ME	Egg 1	0	0	0	0	0	0	0	0	0	0
	Egg 2	0	0	0	0	0	0	0	0	0	
	Egg 3	0	0	0	0	0	0	0	0	0	
Sirolimus ME	Egg 1	0	0	0	0	0	0	0	0	0	
	Egg 2	0	0	0	0	0	0	0	0	0	
	Egg 3	0	0	0	0	0	0	0	0	0	

## Data Availability

All original data are available from the corresponding author on request with a proper reason.

## Abbreviations

The following abbreviations are used in this manuscript:

ALA	Alpha-linolenic acid
AMD	Age-related macular degeneration
BRB	Blood-retinal barrier
COX-2	Cyclooxygenase-2
DR	Diabetic retinopathy
HET-CAM	Hen’s egg test–chorioallantoic membrane
IC50	Inhibitory concentration 50%
ICAM-1	Intercellular adhesion molecule-1
IL-6	Interleukin-6
iNOS	Inducible nitric oxide synthase
LPS	Lipopolysaccharide
LA	Linoleic acid
ME	Microemulsion
NaOH	Sodium hydroxide
NaCl	Sodium chloride
mTOR	Mammalian target of rapamycin
NF-Kb	Necrosis factor-kappa B
S_mix_	Surfactant mixture
TEM	Transmission electron microscopy
TNF-α	Tumor necrosis factor-α
VEGF	Vascular endothelial growth factor
PDGF	Platelet-derived growth factor
PGs	Prostaglandins

## References

[1] Zhang J., Zhang J., Zhang C., Zhang J., Gu L., Luo D., Qiu Q. (2022). Diabetic macular edema: Current understanding, molecular mechanisms and therapeutic implications. Cells.

[2] Heesterbeek T.J., Lorés-Motta L., Hoyng C.B., Lechanteur Y.T.E., den Hollander A.I. (2020). Risk factors for progression of age-related macular degeneration. Ophthalmic Physiol. Opt..

[3] Bosma E.K., van Noorden C.J.F., Schlingemann R.O., Klaassen I. (2018). The role of plasmalemma vesicle-associated protein in pathological breakdown of blood–brain and blood–retinal barriers: Potential novel therapeutic target for cerebral edema and diabetic macular edema. Fluids Barriers CNS.

[4] Al Sakini A.S., Hamid A.K., Alkhuzaie Z.A., Al-Aish S.T., Al-Zubaidi S., Tayem A.A., Alobi M.A., Al Sakini A.S., Al-Aish R.T., Al-Shami K. (2024). Diabetic macular edema (DME): Dissecting pathogenesis, prognostication, diagnostic modalities along with current and futuristic therapeutic insights. Int. J. Retin. Vitr..

[5] Shyam M., Sidharth S., Veronica A., Jagannathan L., Srirangan P., Radhakrishnan V., Sabina E.P. (2025). Diabetic retinopathy: A comprehensive review of pathophysiology and emerging treatments. Mol. Biol. Rep..

[6] Funatsu H., Noma H., Mimura T., Eguchi S., Hori S. (2009). Association of vitreous inflammatory factors with diabetic macular edema. Ophthalmology.

[7] van der Wijk A., Hughes J.M., Klaassen I., Van Noorden C.J.F., Schlingemann R.O. (2017). Is leukostasis a crucial step or epiphenomenon in the pathogenesis of diabetic retinopathy?. J. Leukoc. Biol..

[8] Haydinger C.D., Ferreira L.B., Williams K.A., Smith J.R. (2023). Mechanisms of macular edema. Front. Med..

[9] Vessey K.A., Jobling A.I., Greferath U., Fletcher E.L. (2024). Pharmaceutical therapies targeting autophagy for the treatment of age-related macular degeneration. Curr. Opin. Pharmacol..

[10] Purola P., Kaarniranta K., Ojamo M., Gissler M., Uusitalo H. (2023). Visual impairment due to age-related macular degeneration during 40 years in Finland and the impact of novel therapies. Acta Ophthalmol..

[11] Singh M., Negi R., Alka Vinayagam R., Kang S.G., Shukla P. (2024). Age-related macular degeneration (AMD): Pathophysiology, drug targeting approaches, and recent developments in nanotherapeutics. Medicina.

[12] Wong W.L., Su X., Li X., Cheung C.M.G., Klein R., Cheng C.-Y., Wong T.Y. (2014). Global prevalence of age-related macular degeneration and disease burden projection for 2020 and 2040: A systematic review and meta-analysis. Lancet Glob. Health.

[13] Liao Z.-Y., Hung C.-Y., Hsu Y.-J., Liang I.-C., Chen Y.-C., Sung C.-H., Hung C.-F. (2025). Phlorizin Protects Against Oxidative Stress and Inflammation in Age-Related Macular Degeneration Model. Biomolecules.

[14] Wang J., Han J., Wang X., Han W. (2025). The global burden and attributable risk factor analysis of age-related macular degeneration in 204 countries and territories, 1990–2021. Eye.

[15] Karkhaneh R., Faghihi H., Riazi-Esfahani H., Abrishami M., Bazvand F., Ebrahimiadib N., Johari M., Shoeibi N., Nowroozzadeh M.H., Astaneh M.R.A. (2024). Evaluating the efficacy and safety of aflibercept biosimilar (P041) compared with originator product in patients with neovascular age-related macular degeneration. Ophthalmol. Retin..

[16] Luaces-Rodriguez A., Mondelo-Garcia C., Zarra-Ferro I., Gonzalez-Barcia M., Aguiar P., Fernandez-Ferreiro A., Otero-Espinar F.J. (2020). Intravitreal anti-VEGF drug delivery systems for age-related macular degeneration. Int. J. Pharm..

[17] Callan A., Heckman J., Tah G., Lopez S., Valdez L., Tsin A. (2025). VEGF in Diabetic Retinopathy and Age-Related Macular Degeneration. Int. J. Mol. Sci..

[18] Sarkar A., Sodha S.J., Junnuthula V., Kolimi P., Dyawanapelly S. (2022). Novel and investigational therapies for wet and dry age-related macular degeneration. Drug Discov. Today.

[19] Gomez-Lumbreras A., Ghule P., Panchal R., Giannouchos T., Lockhart C.M., Brixner D. (2023). Real-world evidence in the use of Bevacizumab in age-related macular degeneration (ArMD): A scoping review. Int. Ophthalmol..

[20] Ghasemi Falavarjani K., Nguyen Q.D. (2013). Adverse events and complications associated with intravitreal injection of anti-VEGF agents: A review of literature. Eye.

[21] Ng A., Woods J., Jahn T., Jones L.W., Ritter J.S. (2022). Effect of a novel omega-3 and omega-6 fatty acid supplement on dry eye disease: A 3-month randomized controlled trial. Optom. Vis. Sci..

[22] Zhu W., Wu Y., Li G., Wang J., Li X. (2014). Efficacy of polyunsaturated fatty acids for dry eye syndrome: A meta-analysis of randomized controlled trials. Nutr. Rev..

[23] Giannaccare G., Pellegrini M., Sebastiani S., Bernabei F., Roda M., Taroni L., Versura P., Campos E.C. (2019). Efficacy of omega-3 fatty acid supplementation for treatment of dry eye disease: A meta-analysis of randomized clinical trials. Cornea.

[24] Rashid S., Jin Y., Ecoiffier T., Barabino S., Schaumberg D.A., Dana M.R. (2008). Topical omega-3 and omega-6 fatty acids for treatment of dry eye. Arch. Ophthalmol..

[25] Li Z., Choi J.-H., Oh H.-J., Park S.-H., Lee J.-B., Yoon K.C. (2014). Effects of eye drops containing a mixture of omega-3 essential fatty acids and hyaluronic acid on the ocular surface in desiccating stress-induced murine dry eye. Curr. Eye Res..

[26] Barabino S., Horwath-Winter J., Messmer E.M., Rolando M., Aragona P., Kinoshita S. (2017). The role of systemic and topical fatty acids for dry eye treatment. Prog. Retin. Eye Res..

[27] Yellepeddi V.K., Palakurthi S. (2016). Recent advances in topical ocular drug delivery. J. Ocul. Pharmacol. Ther..

[28] Kumar R., Sinha V.R. (2014). Preparation and optimization of voriconazole microemulsion for ocular delivery. Colloids Surf. B Biointerfaces.

[29] Mahran A., Ismail S., Allam A.A. (2021). Development of triamcinolone acetonide-loaded microemulsion as a prospective ophthalmic delivery system for treatment of uveitis: In vitro and in vivo evaluation. Pharmaceutics.

[30] Amini M.A., Karbasi A., Vahabirad M., Khanaghaei M., Alizamir A. (2023). Mechanistic insight into age-related macular degeneration (AMD): Anatomy, epidemiology, genetics, pathogenesis, prevention, implications, and treatment strategies to pace AMD management. Chonnam Med. J..

[31] Shi J., Yang J., Xu H., Luo Q., Sun J., Zhang Y., Liang Z., Zhao N., Zhang J. (2023). Preparation of a Sunitinib loaded microemulsion for ocular delivery and evaluation for the treatment of corneal neovascularization in vitro and in vivo. Front. Pharmacol..

[32] Tripathi C.B., Gupta N., Kumar P., Singh A.K., Raj V., Parashar P., Singh M., Kanoujia J., Arya M., Saraf S.A. (2018). ω-3 Fatty acid synergized novel nanoemulsifying system for rosuvastatin delivery: In vitro and in vivo evaluation. AAPS PharmSciTech.

[33] Kang S.G., Lee G.B., Vinayagam R., Do G.S., Oh S.Y., Yang S.J., Kwon J.B., Singh M. (2022). Anti-inflammatory, antioxidative, and nitric oxide-scavenging activities of a quercetin nanosuspension with polyethylene glycol in LPS-induced RAW 264.7 macrophages. Molecules.

[34] Zhu X., Wang B., Zhang X., Chen X., Zhu J., Zou Y., Li J. (2020). Alpha-linolenic acid protects against lipopolysaccharide-induced acute lung injury through anti-inflammatory and anti-oxidative pathways. Microb. Pathog..

[35] Lee G.B., Kim Y., Lee K.E., Vinayagam R., Singh M., Kang S.G. (2024). Anti-inflammatory effects of quercetin, rutin, and troxerutin result from the inhibition of NO production and the reduction of COX-2 levels in RAW 264.7 cells treated with LPS. Appl. Biochem. Biotechnol..

[36] Stanca H.T. (2024). Age-Related Macular Degeneration (AMD). Clinical Ophthalmology: A Guide to Diagnosis and Treatment.

[37] Parmar U.P.S., Surico P.L., Mori T., Singh R.B., Cutrupi F., Premkishore P., Afflitto G.G., Di Zazzo A., Coassin M., Romano F. (2025). Antioxidants in age-related macular degeneration: Lights and shadows. Antioxidants.

[38] Singh M., Bharadwaj S., Lee K.E., Kang S.G. (2020). Therapeutic nanoemulsions in ophthalmic drug administration: Concept in formulations and characterization techniques for ocular drug delivery. J. Control. Release.

[39] Singh M., Kanoujia J., Singh P., Tripathi C.B., Arya M., Parashar P., Sinha V.R., Saraf S.A. (2016). Development of an α-linolenic acid containing soft nanocarrier for oral delivery: In vitro and in vivo evaluation. RSC Adv..

[40] Wang Y., Zhou K., Ye C., Shang Y., Wang F. (2025). Preparation and characterization of microemulsions formulated with PEG fatty acid glycerides. Colloids Surf. B Biointerfaces.

[41] Ma W., Du N., Hou W. (2025). Thermodynamic explanation of surfactant-free microemulsions. J. Phys. Chem. B.

[42] Singh M., Kanoujia J., Parashar P., Arya M., Tripathi C.B., Sinha V.R., Saraf S.K., Saraf S.A. (2018). Augmented bioavailability of felodipine through an α-linolenic acid-based microemulsion. Drug Deliv. Transl. Res..

[43] López-Alemany A., Montés-Micó R., Garcia-Valldecabres M. (1999). Ocular physiology and artificial tears. J. Am. Optom. Assoc..

[44] Radomska-Soukharev A., Wojciechowska J. (2005). Microemulsions as potential ocular drug delivery systems: Phase diagrams and physical properties depending on ingredients. Acta Pol. Pharm..

[45] Suri R., Neupane Y.R., Mehra N., Nematullah M., Khan F., Alam O., Iqubal A., Jain G.K., Kohli K. (2021). Sirolimus loaded chitosan functionalized poly (lactic-co-glycolic acid)(PLGA) nanoparticles for potential treatment of age-related macular degeneration. Int. J. Biol. Macromol..

[46] Zafar S., Akhter S., Ahmad I., Hafeez Z., Rizvi M.M.A., Jain G.K., Ahmad F.J. (2020). Improved chemotherapeutic efficacy against resistant human breast cancer cells with co-delivery of Docetaxel and Thymoquinone by Chitosan grafted lipid nanocapsules: Formulation optimization, in vitro and in vivo studies. Colloids Surf. B Biointerfaces.

[47] Narvekar P., Bhatt P., Fnu G., Sutariya V. (2019). Axitinib-loaded poly (lactic-co-glycolic acid) nanoparticles for age-related macular degeneration: Formulation development and in vitro characterization. Assay. Drug Dev. Technol..

[48] Stahl A., Paschek L., Martin G., Gross N.J., Feltgen N., Hansen L.L., Agostini H. (2008). Rapamycin reduces VEGF expression in retinal pigment epithelium (RPE) and inhibits RPE-induced sprouting angiogenesis in vitro. FEBS Lett..

[49] Haeri A., Osouli M., Bayat F., Alavi S., Dadashzadeh S. (2018). Nanomedicine approaches for sirolimus delivery: A review of pharmaceutical properties and preclinical studies. Artif. Cells Nanomed. Biotechnol..

[50] Chatzitaki A.-T., Jesus S., Karavasili C., Andreadis D., Fatouros D.G., Borges O. (2020). Chitosan-coated PLGA nanoparticles for the nasal delivery of ropinirole hydrochloride: In vitro and ex vivo evaluation of efficacy and safety. Int. J. Pharm..

[51] Anand R., Kaithwas G. (2014). Anti-inflammatory potential of alpha-linolenic acid mediated through selective COX inhibition: Computational and experimental data. Inflammation.

